# Pharmaceutical information systems and possible implementations of informed consent - developing an heuristic

**DOI:** 10.1186/1472-6939-13-30

**Published:** 2012-11-16

**Authors:** Thomas Ploug, Søren Holm

**Affiliations:** 1 Centre for Applied Ethics and Philosophy of Science, Department of Communication, Aalborg University Copenhagen, A. C. Meyers Vænge, 2450, København SV, Denmark; 2 University of Manchester, Centre for Social Ethics and Policy, School of Law, Manchester M13 9PL, United Kingdom; 3 Center for Medical Ethics, Faculty of Medicine, University of Oslo, Oslo, Norway

**Keywords:** Autonomy, Control, Health information, Health information systems, Informed consent, Routinisation

## Abstract

**Background:**

Denmark has implemented a comprehensive, nationwide pharmaceutical information system, and this system has been evaluated by the Danish Council of Ethics. The system can be seen as an exemplar of a comprehensive health information system for clinical use.

**Analysis:**

The paper analyses 1) how informed consent can be implemented in the system and how different implementations create different impacts on autonomy and control of information, and 2) arguments directed towards justifying not seeking informed consent in this context.

**Results and Conclusion:**

Based on the analysis a heuristic is provided which enables a ranking and estimation of the impact on autonomy and control of information of different options for consent to entry of data into the system and use of data from the system.

The danger of routinisation of consent is identified.

The Danish pharmaceutical information system raises issues in relation to autonomy and control of information, issues that will also occur in relation to other similar comprehensive health information systems. Some of these issues are well understood and their impact can be judged using the heuristic which is provided. More research is, however needed in relation to routinisation of consent.

## Background

### Information systems

Information systems holding data on all patients in a given health care system are being developed worldwide. The most ambitious plans envisage a system holding the complete patient record with all attendant data (lab results, imaging data, genetic data etc.) within the system, but such a system has not yet been implemented in any large scale health care system. Such information systems raise many ethical and legal issues. In the present paper we analyse some of these issues, especially issues concerning informed consent. The starting point is a nationwide pharmaceutical information system that has already been implemented in Denmark. By focusing on a concrete, already existing system we hope to be able to provide an analysis based more in clinical and administrative reality than in speculation about the future.

There is a substantial literature on the use of routinely collected patient data for research purposes, but the literature on the ethical issues raised by the collection and use of comprehensive patient data for treatment and diagnostic purposes is more limited. The so far unsuccessful efforts of the English National Health Service to share patient information electronically across all services has generated some ethical debate, [1-4] as has similar initiatives in other countries [5]. In this paper we hope to deepen this debate by showing that issues of consent in this context may be considerably more complicated than they may initially appear. There are many differences between the research context and the clinical context and many of them are relevant in relation to the kind of information systems we analyse here. Although the information in these systems is very useful for research purposes, the primary stated aim of implementing the systems is not to provide knowledge, or to provide general public benefit, but to provide direct benefit to individual patients. These systems involve people’s health related interests much more directly than most research databases do. Another very important difference is that the main purpose of implementing the system necessarily involves using the information it contains, and the inferences that can be drawn from this information in a person-identifiable way. Anonymity is not an option and privacy interests can therefore not be protected by anonymising. This entails that some of the analyses provided in the literature on research databases may not be transferable to the present, clinical context.

The paper falls in 3 sections. We first briefly describe the comprehensive Danish pharmaceutical system and the reasons given for implementing it. In the second part we analyse issues of autonomy and informed consent raised by the introduction of the system and provide a heuristic for classifying these issues in a three dimensional space of relevant considerations. In this second part of the paper we also discuss some arguments raised by the Danish Council of Ethics and show how they can be encompassed by our heuristic. The final part of the paper then considers whether the tension between respect for autonomy and the reasons for introducing the system generate a true ethical dilemma, and discuss further problems created by a possible routinisation of informed consent.

The paper provides a heuristic for analysing these issues which is of value for anyone who thinks that autonomy is important, because the heuristic ranks possible models for informed consent in relation to their impact on autonomy. We do not purport to ‘solve’ the ethical issues since that would require a determination of exactly how important personal autonomy is in relation to all other relevant ethical considerations and values. Such a determination is outside the scope of this paper. Our heuristic is never the less of value, since it provides a detailed account of the effect of different consent arrangements on one side of the balancing between autonomy and other considerations. This should allow decision-makers to have a clearer view of what they are sacrificing in choosing consent arrangements that do not fully protect autonomy.

### The shared medicine profile

Since the passing of the law on personal, electronic, medicine profiles in 2003, the Danish Medicines Agency – an agency under the Ministry of Interior and Health – has registered the personal “use” of prescription medicine among Danes on the basis of reports from all Danish pharmacies (see below for a discussion of “use”) [6,7]. The resulting database feeds information into two separate personal records, the Medicine Profile and the Shared Medicine Profile.

The Medicine Profile is an electronic record containing information about the medicine prescribed for and bought by an individual within the last two years. Medicine prescribed or given during in-patient stays is also registered. The registration is automatic and compulsory. When the law was passed in 2003, pharmacists, doctors and their assistants, the patient and the Danish Medicine Agency were granted access to the records. The Medicine Profile was introduced with the primary purpose of providing doctors, patients and pharmacies with an overview of the medication prescribed for or given to an individual thereby enabling an improved and consistent use of medication leading to benefit for the individual and a reduction in public spending [6,8]. The Medicine Profile was implemented in 2004.

The Shared Medicine Profile is an electronic record collecting and keeping up to date information about a patient’s medical treatment within the last two years across electronic health records in hospitals and general practitioner practices, electronic municipal records of care for the elderly and the Medicine Profile. The Shared Medicine Profile thus contains information about all prescribed medication and any medication provided by health care personnel along with information about the indication on the basis of which the medication is prescribed or provided. It also includes information about any medication bought by the individual apart from over-the-counter medication, the prescribed daily intake and the date of termination of the medical treatment. The health care personnel’s’ instructions of use are also registered along with notes concerning any known non-adherence to treatment, intolerance or allergy. Patients can also themselves register the use of any non-prescription medication through their electronic access to the Medicine Profile. The Medicine Profile and the Shared Medicine Profile both attempt to provide a record of the medication that is used by a particular patient, but because the vast majority of use in the community is unsupervised there will be a, potentially significant discrepancy between what is prescribed and bought and what is actually used. This is only partly alleviated by the possibility of patients themselves adding information to the Shared Medicine Profile, so the official claim that the two profiles show medication “use” is somewhat dubious. The Shared Medicine Profile has further extended the groups of health care personnel with access to the patient’s medication records. Access is now given to doctors, nurses, midwives, health visitors, social and health care assistants, workers in care for the elderly, dentists, certain pharmacists employed at hospitals as well as pharmacists and pharmacy assistants in community pharmacies, as well as staff at The National Board of Health and the Medicines Agency. Subject to the Ministry of Health issuing more specific rules and regulations the access to the Shared Medicine Profile may also in the future be extended to other persons directly involved in distributing medications to individuals in their homes.

The Shared Medicine Profile was introduced with the explicit purpose of sharing information about a patient’s medication between hitherto separated information systems and between health care personnel in different settings. And the aim is to achieve better coordination of medication within and across sectors, faster correction of medication errors,early identification and consideration of potential drug interactions, and a more effective use of health care resources through reducing multiple, identical entries in multiple parallel information systems [7]. The Shared Medicine Profile will be fully implemented by the end of 2012.

## Analysis – The shared medicine profile and informed consent

### The ethical challenge – beneficence, justice and autonomy

The Shared Medicine Profile poses an ethical challenge – perhaps even a dilemma. On the one hand, principles of beneficence and non-maleficence may be taken to imply the moral goodness of actions aimed at the prevention of harm to others [9]. In this case the harms caused by medication errors and drug interactions. Similarly a principle of justice may support actions aimed at distributing health care resources without waste [9]. In this case by reducing unnecessary prescribing and duplication of work by health care personnel. On the other hand, the principle of respect for autonomy is typically understood to imply a requirement to obtain informed consent before intervening in a patient’s life (cf below) [9]. The Shared Medicine Profile represents an intervention in the patient’s life. It involves the collecting and storing of personal health information, and the distribution of the information to a very large group of people. The ethical challenge faced in considering the implementation of a comprehensive health information system such as the Shared Medicine Profile is therefore to balance efforts to effectively gather and disseminate information in order to prevent harm to the individual and promote a just distribution of health care resources against proper respect for the patient’s autonomy.

### Personal autonomy, respect and informed consent

Personal autonomy is associated with the ability to rule or govern oneself, i.e. self-rule and self-government [10]. For present purposes, we shall assume that personal autonomy involves two basic abilities: [11] 1) the ability to exercise one’s cognitive capabilities in a rational formation of and identification with specific goals, values, aims, desires and plans etc., [12-14] and 2) the ability to pursue or implement these goals, values, aims, desires and plans in action without the choice of action being restrained by forces alien to oneself [15,16]. In short, personal autonomy involves the ability to rationally form one’s own goals, values, plans etc., and to pursue these without being restrained in various ways.

Furthermore, we shall assume that *protecting* personal autonomy implies a requirement for a person to take steps to ascertain that a given intervention into another persons’s life does not alienate the person from his or her goals, values and plans, and a requirement not to restrain the person in his or her pursuit of these goals, values and plans. That is, we take it that protecting personal autonomy requires that informed consent is obtained before an intervention into another person’s life. Finally, we shall assume that *promoting* personal autonomy is a matter of empowering a person to rationally form and pursue goals, values and plans unrestrained. Promoting personal autonomy is a matter of providing a person with the power and the opportunity to control his or her life in formation and pursuit of goals, values and plans. Note, very importantly, that although the protection of personal autonomy may be claimed to be in principle acquired through obtaining informed consent, the specific way in which the requirement is implemented may promote personal autonomy to a varying degree.

In the following we will show that the *content* and *presentation* of a particular request for informed consent may provide a person with control over his or her life to very different degrees. The implicit claim is that the protection of personal autonomy through informed consent cannot be separated from the degree to which the personal autonomy is promoted through the content and presentation of the informed consent. Consequently, in considering possible models of informed consent in relation to the implementation of the Shared Medicine Profile we will speak of ’respect for personal autonomy’ as being expressed through the requirement of informed consent, and that therefore – because informed consent both protects and promotes personal autonomy – the ’respect for autonomy’ expressed in the different models of informed consent may vary in degree. In effect we will speak of models of informed consent as expressing a weaker or stronger ’respect for’ or, in this wider sense, ’protection of’ personal autonomy.

### The shared medicine profile and models of informed consent – developing an heuristic

The implementation of the Shared Medicine Profile may incorporate a requirement of informed consent in several ways. The exact requirements for a valid informed consent are a matter of serious philosophical dispute and vary legally between different jurisdictions. In the following we will assume that to be valid informed consent requires that the person providing consent is adequately informed about his options and their consequences, and that consent is provided without undue influence. We believe that this is a rather minimal set of requirements. There are a number of features and aspects of the implementation and use of the Shared Medicine Profile that may be included in the content of an informed consent. Let us call these features and aspects ‘variables’ and the options for each variable ‘values’. The listing of ‘values’ for each ‘variable’ is not intended to be exhaustive but identifies major options:

1. The *registration* of personal information about medication, e.g. consent to:

a. The registration of every prescribed medication

b. The registration of every type of prescribed medication

c. The registration of all prescribed medication

2. The *use* of personal information about medication, e.g. consent to:

a. The possible use of every single piece of medication information

b. The possible use of every type of medication information

c. The possible use of all medication information

3. The *access* to personal information about medication, e.g. consent to:

a. The access to medication information for every individual member of the health personnel (e.g. an individual doctor)

b. The access to medication information for groups of health personnel (e.g. doctors)

c. The access to medication information for all health personnel (and potentially others)

4. The *exchange* of personal medication information between medical information systems, e.g. consent to:

a. Every exchange of health information between medical information systems

b. Exchange of health information between medical information systems within the same sector

c. Exchange of health information between all health information systems

5. The *authority* to extend access to personal medication information to new groups of health personnel, e.g. consent to:

a. The authority to extend access to one person (e.g. Minister of Health)

b. The authority to extend access to a specific group of persons (e.g. medical doctors)

c. The authority to extend access to several groups and individuals.

The above variables of informed consent are all variables relating to the content of the informed consent. They concern what the patient is ‘’ consenting to’. The different possible values of these variables provide the patient with more or less control over the intervention in the patient’s life caused by the implementation of the Shared Medicine Profile. Assuming that respecting personal autonomy involves providing the patient with control over interventions, it follows that the inclusion of specific values for each of these variables in the informed consent process may either, relatively speaking, strengthen or weaken the protection of personal autonomy. For example, if the patient consents to the access to medication information for all health personnel then he or she will in the future not be able to control the access to the information as effectively, as if he or she had restricted the availability to only some groups. This means that the patient will not, for instance, be able to restrict access to sensitive information only to health care personnel that he or she trusts. This clearly weakens the protection of personal autonomy. It is important to note that we are not claiming that increased control over information is necessarily welfare maximising. A person may clearly use this control contrary to his or her own interests. What we are claiming is that increased control over personal information increases personal autonomy. This is, we submit, close to an analytical truth.

In the following we will reduce these five variables to two, ‘registration’ and ‘use’. The reasons for this are primarily: 1) That ‘access’, ‘exchange’ and ‘authority to change’ can be seen as aspects of ‘use’ of information, e.g. that a restriction to a particular group of health care personnel is a restriction of a specific use of the medication information, and 2) that in the Danish context the Shared Medicine Profile operates within a predominantly public health care system with clear lines of political accountability. Issues concerning ‘exchange’ and ‘authority to change’ are thus less prominent.

There are, however, other features of the Shared Medicine Profile that may influence the strength of the protection of personal autonomy through the informed consent process. In general, the way in which information is presented or provided will influence an individual’s possibility of making informed choices, and therefore also influence the individual’s ability to control his or her life in accordance with the individual’s own goals and values. The validity of informed consent relies on the patient having been adequately informed and having understood this information. The likelihood of this being attained depends on a large number of factors including an appropriate amount of information being given, the information being of high quality and relevance, and the information being delivered in a way that maximises the possibility that the patient will understand the information [17-20]. Information about the Shared Medicine Profile may be disseminated in various ways, and we will assume that the likelihood of adequate understanding increases if the information is delivered in a personal interaction. And we will assume, that very general information campaigns are likely to be the least effective. We thus have a composite variable reflecting the likelihood of good understanding of the information provided (we assume that the information provided is in all circumstances adequate and of high quality):

6. The adequacy of the information process, e.g.

a. The information is provided personally by health personnel as part of the informed consent process

b. The information is provided through leaflets distributed by health personnel

c. The information is provided through general information campaigns

Personal interaction increases the possibility that a health care professional may exert undue influence on a patient’s choice. We will in the following assume that this risk can be mitigated by professional regulation, and that it is overall more likely that a valid informed consent takes place when there is personal interaction as part of the informed consent process.

This list of variables and their possible values is not exhaustive. They represent an attempt to capture some of the features and aspects of the Shared Medicine Profile important for the informed consent process. Other variables could have been taken into account. Thus there is a longstanding discussion of the exact informational requirements for valid informed consent [21-26]. This is not unimportant but we are here focusing on how the more specific features and aspects of the Shared Medicine Profile are relevant to informed consent.

We have so far identified three fundamental variables – ‘registration’, ‘use’ and ‘adequacy of information’ – relevant for the implementation of comprehensive health information systems such as the Shared Medicine Profile. Although the list of variables and their possible values may not be exhaustive, there is a particular reason for the inclusion of these variables and these values. Thus it seems to hold that the degree of protection of personal autonomy provided by choosing certain variables and certain values logically dominates the protection provided by certain other sets of variables and values, where one model of informed consent, say *A*, logically dominates another, say *B*, if and only if the protection of personal autonomy entailed by *B* is fully included in *A*, i.e. if *A* provides a protection of personal autonomy which is greater than *B*. In other words, the patient’s control over interventions into his or her informational domain based on agreeing to some of these variables and values through informed consent dominates the control over such interventions based on choosing another set of these variables and values. This is easily seen. Thus the control gained if the patient has to give informed consent to every registration (1.a) and every use of information (2.a), clearly dominates the control gained by only having to give a general consent to registration (1.c) and use (2.c) of information. In fact, it seems as if the combination of requiring consent to both the registration (1.a) and use (2.a) of every piece of medication information provides a degree of control over personal medication information that dominates the control gained by having to negotiate an informed consent based on any of the remaining variables and values (1.b-c, 2.b-c, 3.a-5.c). This account of domination entails that whereas there are combinations of values that dominate other combinations of values, there are also combinations of values where one set does not clearly dominate another set (cf below); or more formally that the ranking of value 3-tuples is not complete.

The protection of autonomy gained by requiring informed consent based on the last variable and it’s possible values (6.a-b) is not implied by the combined requirement of informed consent (1.a and 2.a). This is hardly surprising since this variable does not concern what the patient is consenting to, but rather the way in which the patient is being informed about the workings of the Shared Medicine Profile.

### An attempt at an ethical heuristic

**Figure 1:**
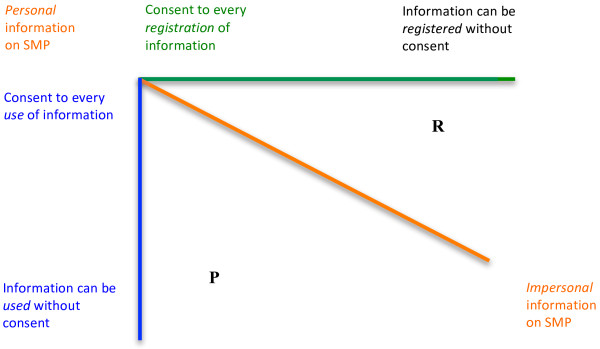
**The three-dimensional space of informed consent**.

The three variables identified above are fundamental in the sense that if given particular values and incorporated into a requirement of informed consent, they will express the strongest possible protection of personal autonomy. Since these variables may take different values, the many possible constructions of informed consent constituted by different values of the variables may be expressed by letting each of the variables be constituted by an axis in a three-dimensional space (Figure 1).

The three-dimensional space illustrates the various possibilities of constructing a requirement of informed consent, but also raises the question of how the different points in this space are to be ranked. At the point of the intersection of the three axes, the protection of personal autonomy through informed consent is evidently strongest. It is less evident, however, how, for instance, the point ***P*** is to be ranked in comparison to the point ***R***.

**Figure 2:**
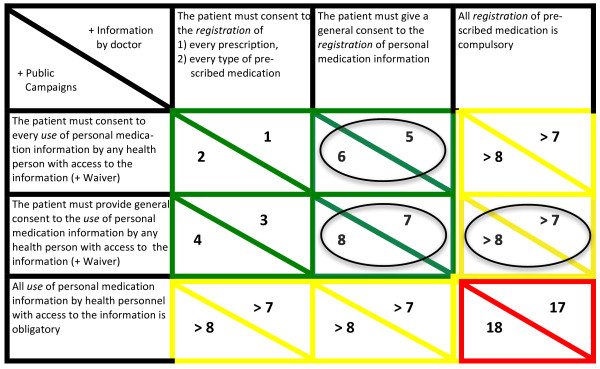
**Models of informed consent, Figure**2**consists of the three-dimensional space of Figure**1**with the addition of well-defined points on the ’registration-axis’ (horisontal), the ’use-axis’ (vertical), and the ’information-axis’.** The numbers give the ranking of the specific point (i.e. triangle) in the three-dimensional space in which it is positioned such that the higher the number the weaker the protection of personal autonomy. The colours mark three basic clusters of points according to their protection of personal autonomy. The circles mark the ’positions’ taken by members of the Danish Council of Ethics.

Instead of a generalized consideration of the relative strength of the protection of personal autonomy at these points in the space of values, it seems more interesting in terms of policy considerations to consider more well-defined points on each of the axes. To illustrate such well-defined points we shall here reconstruct the positions taken by the members of the Danish Council of Ethics in the Council’s report on the Shared Medicine Profile. In the matrix below three well-defined points on both the *registration*- and *use*-axes have been picked out together with two points on the *information*-axis (Figure 2).

The matrix contains 18 fields. The circled fields mark the recommendations of members in the Danish Council of Ethics. (The Danish Council of Ethics is not required to reach consensus among its members before making recommendations). The coordinates on the *use*-axis all state that consent is to the use ‘by any personnel with access to the information’. This amendment is intended to express the view, taken by all members of the Danish Council of Ethics, that only certain groups of health care personnel should have access to the medication information. In itself the limitation does not strengthen the protection of personal autonomy. If the patient is to provide consent to any use of medication information, then logically speaking it does not matter if the group of people covered by the consent is given is limited or not. The amendment could be said, however, to strengthen the protection of personal autonomy when considering waivers to the requirement of obtaining consent before use of personal medication information. All members of the Danish Council of Ethics agree that there are situations in which the requirement of obtaining informed consent before use of personal medication information is trumped by other ethical concerns [8]. In those situations, limitations on the groups of people with access to the information could be said to limit or reduce the loss of control over access to the information, and in this sense the limitation implies a strengthening of the protection of personal autonomy as compared to having no limitations.

The Danish Council of Ethics makes three recommendations concerning the registration and use of information. The Council takes two different views on the registration of personal medication information [8]. One group recommends that the patient is given the right to refuse the registration of personal medication information, i.e. that one-off informed consent must be provided before the registration of personal medication information. Another group recommends that the registration should be compulsory. On the use of the information the Council members also takes two different views [8]. One group recommends that the patient is to provide consent on every occasion of personal medication information being retrieved and used. Another group recommends that a one-off informed consent is provided to the use of personal medication information to health care personnel other than the patient’s own general practitioner and The National Board of Health. Given the different views on the registration and use we thus end up with the three recommendations of the Council marked by circles in the matrix.

Of special importance is the ranking of each of the fields. The fields are ranked according to the degree of protection of personal autonomy provided. The ranking is marked by both numbers and colouring. The numbers mark the ranking of the relevant field such that the higher the number the weaker the protection of personal autonomy. The reasoning behind this ranking is key to fully understanding the matrix and it’s implications – and, as we shall see, the reasoning also on a more general level deepens our understanding of how personal autonomy may be interpreted and protected when implementing information systems such as the Shared Medicine Profile.

The weakest protection of personal autonomy – arguably no protection of personal autonomy at all – occurs when both the registration and use of personal medication information is compulsory and takes place merely on the basis of general, impersonal information about the Shared Medicine Profile. The ranking as 18 is thus straightforward. The ranking as 17 is less evident. However, it seems reasonable to claim that the relevant ways in which a patient is informed about the Shared Medicine Profile is less important to the protection of autonomy than being offered the choice of consenting to the registration or to the use of personal medication information as long as the information about the Shared Medicine Profile is available to the patient, i.e. as long as it is *possible* to make an *informed* choice. The choice of giving or refusing consent allows the patient to control the intervention of others into the life of the patient, whereas the relevant ways in which information about the Shared Medicine Profile is provided affects the degree to which the patient is able to make an informed choice. From these considerations it follows more generally that the difference between informing the patient through public campaigns and through personal encounters with health care professionals in itself implies a difference in ranking of only one place. This explains the difference in ranking between 1 and 2, 3 and 4, and so on – and therefore also in turn the difference between 17 and 18. (Note that in the special cases of the fields 17 and 18 the difference in ranking is also partly explained by the fact that the very act of informing a patient about compulsory registration provides the patient with an opportunity to avoid doctors or to avoid prescriptions. Hence the information provides a patient with an opportunity to exercise a very limited kind of control).

This line of reasoning could be questioned on various grounds. It may be argued that the way in which information is provided carries different weight depending on the variant of informed consent, i.e. field, under consideration. Thus one could claim that the fields currently ranked <1, 2, 3, 4 > should be ranked <1, 3, 2, 4> with the other rankings remaining the same. However, we shall maintain the current ranking – not least because it seems that valid consent before the registration or use of personal information in practice simply cannot be obtained without providing considerable information about the Shared Medicine Profile. Thus the theoretical difference between a health care professional personally informing a patient and information only being provided by public campaigns seems to be reduced significantly when considering the practical circumstances of having to obtain consent.

The additional rankings are based on a number of considerations. As discussed above, informed consent is supposed to be a way of protecting an individual’s ability to form and pursue his or her own plans and goals. It seems to follow that any construction of informed consent that will extend both the available ways of controlling the influence of other’s on the pursuit of goals and plans but also, more generally, extend the available courses of action is preferable. The possible models of informed consent found in the matrix provide different degrees of control and possibilities for pursuing the benefits associated with the Shared Medicine Profile. Those found in the fields <1,2>, <3,4>, <5,6> and <7,8> − here paired on the basis of the same coordinates on the registration- and use-axes and referred to by their ranking – all provide the patient with some control over the registration and use of information while at the same time making it possible for the patient to achieve the potential benefits of the implementation of the Shared Medicine Profile, i.e. a better coordination of medication within and across sectors, a faster correction of medication errors and an earlier identification of potential drug interactions.

In the model of informed consent found in the fields <1,2> the patient is given control over the registration of every piece or type of personal medication information. By controlling every piece or type of medication information the patient is given very flexible control over the information available for use by health personnel, but also and more importantly, the patient is given flexible control over the information potentially abused by health personnel or otherwise lost to third parties. The abuse may happen through people acquiring unauthorized access to the information, but also through health personnel acquiring access by falsely or unjustifiably claiming a situation to be among the waivers where concurrent patient consent is not necessary. (The Danish Medicines Agency has at this point in the implementation process reported on five cases of unauthorized access to the Shared Medicine Profile [8]). In the fields <1,2> the patient is also given control over the use of personal medication information by a particular health person in a particular situation. Again, the control over the use of information by a particular health person in a particular situation makes it a very flexible control. The patient is able to control whom to entrust with personal medication information and when. In total the construction of informed consent found in the fields <1,2> represents a very flexible protection of the patient’s personal autonomy in that it allows the patient to act upon his or her own balancing of the potential benefits and harms from having a specific piece of information registered, and from allowing a specific person to access personal medication information on a particular occasion.

In the model of informed consent found in the fields <3,4> there is a slight weakening of the flexibility in the patient’s control over personal medication information. The patient retains the possibility of weighing benefits and harms associated with having a specific, single piece or type of personal medication information registered, but cannot flexibly control what information a particular person may access on a particular occasion.

In the model of informed consent found in the fields <5,6>, there is also a weakening of the flexibility in the patient’s control. Thus the patient cannot both realise the benefits from registering personal medication information, and at the same time retain control over what information that is registered. It is simply all or nothing. Control over the pool of information that may be used and abused is, in these fields incompatible with the realisation of the benefits from registering personal medication information. However, the patient is given control over the use of personal medication information by a particular person on a particular occasion. The reason for ranking these fields, <5,6>, lower than the previous fields, <3,4> and <1,2> , is simply that the combination of values makes it impossible to both gain benefits from registering personal medication information while at the same time retaining a degree of control over the registration and use of personal medication information. Thus <1,2> and <3,4> provides a stronger protection of personal autonomy than <5,6> in virtue of providing flexible control over information in combination with the additional possibility of realising at least some of the benefits associated with the implementation of the Shared Medicine Profile. This argument in favour of the current ranking of the relevant fields obviously presupposes that it is possible to realise some of the benefits claimed to be associated with the Shared Medicine Profile without having to register all of one’s personal medication information. It seems, however, that better coordination of medication within and across sectors, faster correction of medication errors and earlier identification of potential drug interacttions are benefits that may to some degree be achieved without the registration of all personal medication information (cf. below).

The model of informed consent found in the fields <7,8> implies a further weakening of the control over the use of personal medication information as compared to <5,6>. All other constructions of informed consent found in the matrix involve a weakening of the patient’s control over personal medication information by making either the registration or use of personal medication information compulsory. The ranking of these fields will consequently be above 8 and below 17 – the specific ranking may be worked out on the basis of the considerations found above.

## Discussion

### Limits to the requirement of informed consent

In the previous section we introduced a heuristic for developing and evaluating different possible ways of implementing a requirement of informed consent in relation to the Shared Medicine Profile. Using the heuristic we ranked a number of possible models of informed consent. Assuming the value of personal autonomy, the previous section in effect leaves us with the question of what reasons there are for implementing any model but the one that provides the patient with the strongest protection of his or her personal autonomy, i.e. <1>. As already mentioned, there are possible reasons for implementing a weaker model of informed consent, and it is to the analysis of these we now turn.

Generally speaking any requirement of obtaining informed consent, before the intervention in a patient’s life may be weakened or no longer apply, if one or more of the following is the case:

1) There are insurmountable practical difficulties associated with obtaining consent

2) The patient lacks the competence required to provide consent,

3) There are other ethical concerns trumping the principle of respect for autonomy,

4) The practice of obtaining informed consent in itself undermines the protection of personal autonomy.

It is, for instance, a combination of these considerations that is usually taken to justify the common practice of sharing information without consent within the immediate group of health care personnel involved in the patient’s care. However, the information sharing in relation to the Shared Medicine Profile is much wider and requires separate analysis and justification.

The practical difficulties associated with the implementation of informed consent clearly hinges on the specific model of informed consent. The models providing the strongest protection of personal autonomy require that consent is obtained on many occasions, whereas the models providing the weakest protection only require a single valid consent. The practical difficulties will also depend on the character of the informed consent process. If it is handled as other consent processes in clinical practice then the health care professional must – on the basis of having informed about the Shared Medicine Profile – direct a request to the patient to provide consent to the registration and use of the patient’s personal medication information. This request could alternatively be put to the patient electronically. The Shared Medicine Profile provides the patient with web access to the entries in his or her profile. An electronic request of consent could be transmitted via the Shared Medicine Profile – and in case the consent is given the transfer of information to the profile and between information systems could be automatically initiated. Whichever way the requirement of informed consent is implemented it seems unreasonable to characterise the practical difficulties as insurmountable. Significant, but surmountable practical difficulties cannot as such rule out any implementation of informed consent, although they may be relevant to further ethical considerations of whether or not to prioritise the implementation of a given model of informed consent.

In the opinion of some members of the Danish Council of Ethics the registration and use of personal medication information must be made compulsory for the simple reason “” that many patients will find it difficult to grasp the advantages and disadvantages of not being included in the Shared Medicine Profile. In particular this would hold in relation to the group of patients (…) undergoing treatment with a variety of medicines (…).” [8] In the first general part of this statement the opinion seems to be that people in general do not satisfy standard criteria of decisional competence [27-30] in that they are considered unable to comprehend the consequences for them of being included in the Shared Medicine Profile. Unfortunately there is no further elaboration on what particular features of the Shared Medicine Profile that patients are supposedly, unable to grasp. There seems to be little difference between the nature of the intervention into a patient’s life constituted by the implementation of the Shared Medicine Profile and other medical interventions for which informed consent is required.

As we briefly discussed above, there are strong ethical reasons for implementing the Shared Medicine Profile. SMP improves conditions for providing medical care in situations of emergency, and more generally leads to better coordination of medication within and across sectors, faster correction of medication errors,earlier identification of potential interactions, and reduced waste of resources in the Health Sector. The question remains, however, if the satisfaction of the principles of beneficence and justice in the case of the Shared Medicine Profile are inconsistent with respecting and protecting personal autonomy through the implementation of a requirement of informed consent, i.e. if we are here faced with a true dilemma.

In order for the Shared Medicine Profile to present a true dilemma two mutually intertwined empirical conditions must be satisfied. Firstly, it has to be the case that no single patient can reap the benefits of the Shared Medicine Profile without registering all personal medication information. If there are individual health benefits and reduced waste of resources to be gained from only partial access to the patient’s personal medication information, then the implementation of a requirement of informed consent resulting in a limited registration of information or limited access to use the registered information, will not pose a hindrance to the improvement of health care. In this case then, the implementation of a requirement of informed consent will not be inconsistent with satisfying other principles such as beneficence and justice. The question therefore is, if there are benefits that may be achieved without full access to the patient’s information. It seems that partial access will – at least to some extent – allow for better coordination of medication within and across sectors, faster correction of medication errors, earlier identification of potential interactions, and also lead to reduced waste of the resources going into the parallel development and maintenance of separate information systems in the health care sector. Partial access to personal medication information may be a problem in terms of discovering potential drug interactions solely on the basis of studying medication records in the SMP, but some cases of interactions may be detected on the basis of access to less than complete personal medication information.

The second empirical prerequisite of a true dilemma concerns the patient’s actual behaviour in terms of providing consent. If the implementation of the Shared Medicine Profile is to pose a true dilemma, then we must be able to rule out the possibility that adequately informed patients voluntarily choose to consent to the registration and use of their personal medication information. However, there seems to be little evidence of widespread refusal of consent – at least in a Danish context. On the one hand, it does seem reasonable to expect that some consent processes may result in the withholding of consent. On the other hand, it does not seem as if patients generally exhibit a restrictive behaviour in other consent processes in the health sector. The extent to which consent will be refused is thus hard to predict. The point still applies, however, that if we cannot rule out the possibility that people voluntarily choose to provide consent, then the implementation of the Shared Medicine Profile with a requirement of obtaining informed consent does not necessarily pose a true dilemma.

Although the above considerations of the empirical preconditions of a true dilemma raise some important questions, they also point to a different sense in which the Shared Medicine Profile poses a dilemma. The implementation of the Shared Medicine Profile along with a requirement of informed consent is *de facto* incompatible with the *maximal* satisfaction of the targets of better coordination of medication, faster correction of medication errors,earlier identification of potential drug interactions, and reduced waste of health care resources. In so far as the principles of beneficence and of justice are taken to be requirements of maximal beneficence and justice, then the complete satisfaction of these principles is incompatible with the satisfaction of the requirement of informed consent. If the satisfaction of the principles of beneficence and justice may be gradual or partial, then it clearly is the case that the implementation of the Shared Medicine Profile with a requirement of informed consent is compatible with beneficence and justice.

One might here take the position that both of the empirical conditions are *de facto* satisfied: 1) the benefits of the implementation of the Shared Medicine Profile can only be realised on condition of full access to the personal medication information and patients would generally choose to withhold consent; and 2) that the principles of beneficence and justice are to be the sole principles guiding action. Consequently, the Shared Medicine Profile must be implemented with a requirement of compulsory registration and full access to the use of personal medication information. However attractive this position may seem, it conflicts with the strong tradition in the health care sector of giving priority to the protection of personal autonomy in situations that exhibit a significant similarity with this one. Thus it is common practice to accept a patient’s refusal of therapy that medically is considered to be appropriate, and instead provide therapy that is considered to be less adequate, but in accordance with the goals, plans and values of the patient – even in cases where this may lead to increased public costs. This leaves a proponent of the position sketched above with two options. Either the ethically relevant difference between clinical practice and the implementation of the Shared Medicine Profile has to be pointed out, or one must argue in favour of restricting the protection of personal autonomy through informed consent in all of these situations.

The fourth and final reason for restricting the use of informed consent in relation to the Shared Medicine Profile is if a requirement of informed consent undermines the very function of the informed consent as a protection of personal autonomy. How is that possible? The answer is that the informed consent may lose its function as a protection of personal autonomy if it is routinised. By routinisation is meant the provision of consent as a routine action, i.e. as an unreflected, habitual act. If the provision of consent becomes an act of routine then it will no longer protect the personal autonomy since the very essence of this protection is about reflecting on the consistency of another’s suggested intervention with one’s goals, plans and values. On the assumption that routinisation may undermine the function of informed consent as suggested here, it evidently becomes of interest to clarify if the possible implementations of informed consent discussed in the previous sections may lead to the routinisation of consent. The process of providing consent before installing computer software may be seen as an analogous case shedding some light on this question. It seems to be a common experience that the consent provided before installing software is routinised – the consent is provided as an unreflective, habitual act. The act of providing consent is simply reduced to a number of steps that have to be taken before access to certain attractive functionalities is acquired. In this case the routinisation seems to be conditioned by four factors. First, the unmanageability of the extensive information on the conditions of use. Second, the high frequency with which consent is to be provided. Third the strong desire to acquire the functionalities constituted by the software. Fourth, the expectation that the conditions of use are not unreasonable. The second factor is highly relevant when considering the possible implementation of informed consent discussed previously. If informed consent is required for every registration and use of personal medication information, then a significant number of people will be asked to provide consent rather often. If this model of consent is implemented such that the consent is to be provided to health personnel in the course of treatment, then the third and fourth factor are also relevant. The provision of consent may thus be influenced by a desire to start or focus upon treatment, and it seems likely that many people will expect the actions suggested by the health personnel – access to the patient’s personal medication information – to be in their best interest. The general conclusion therefore seems to be that at least some of the suggested models of informed consent may lead to the routinisation of the provision of informed consent, and hence to the undermining of the ability of the informed consent to protect personal autonomy. It has to be noted, however, that this argument needs further substantiation. Routinisation ultimately refers to an empirical condition, and therefore has to be vindicated as such. Note also, that although the argument from routinisation undermines extensive and repeated use of informed consent in the implementation of the Shared Medicine Profile, it is wholly consistent with the assertion of the principle of respect for personal autonomy. Thus one may hold that it exactly is because personal autonomy is worthy of protection, that routinisation poses a problem.

## Conclusion

In this article we have argued that the implementation of a comprehensive health information system such as the comprehensive, nationwide Danish pharmaceutical information system known as the Shared Medicine Profile poses an ethical challenge, namely the challenge of balancing efforts to effectively gather and disseminate information in order to prevent harm to the individual and promote a just distribution of health care resources while at the same time respecting the patient’s autonomy.

In the face of this challenge we have considered possible ways of implementing a requirement of informed consent in the running of an information system such as the the Shared Medicine Profile. To clarify the way in which different options impact on the protection of personal autonomywe have developed a general, ethical heuristic for:

1) Constructing possible models of informed consent, and

2) Evaluating these models with respect to their impact on personal autonomy

Finally, we have considered four possible strategies for denying a requirement of informed consent in relation to the implementation of pharmaceutical information systems such as the Shared Medicine Profile. In relation to these four strategies we have argued:

1) That there are no insurmountable practical difficulties involved in implementing a requirement of informed consent

2) That it seems unlikely that patients should lack the competence required to provide consent in relation to the registering and use of personal medicine information

3) That the implementation of pharmaceutical information systems do not necessarily poses a ‘strong’ ethical dilemma, and

4) That the implementation of a model of informed consent requiring the patient to provide frequent consent may lead to the routinisation of informed consent

With respect to any future research agenda, the fourth conclusion is of special importance. As argued above the routinisation of informed consent undermines the very ability of informed consent to protect personal autonomy, and it seems as if routinisation poses a serious threat to a significant sub-set of the models of informed consent considered in this article. As far as we know the notion has not received any attention in the literature on medical ethics as well as within the broader framework of ethics and moral philosophy.

## Competing interests

The authors declare that they have no competing interests.

## Authors’ contributions

TP drafted the first version which was then substantially revised with input from SH. Both authors contributed to the writing of the final version, and both approve the final version.

## Pre-publication history

The pre-publication history for this paper can be accessed here:

http://www.biomedcentral.com/1472-6939/13/30/prepub
